# Sleep and critical illness: a review

**DOI:** 10.3389/fmed.2023.1199685

**Published:** 2023-09-19

**Authors:** Erin Eschbach, Jing Wang

**Affiliations:** Division of Pulmonary, Critical Care, and Sleep, Mount Sinai Hospital, New York, NY, United States

**Keywords:** sleep, sleep disturbances, critical illness, ICU – intensive care unit, COVID-19, delirium, circadian disruption

## Abstract

Critical illness and stays in the Intensive Care Unit (ICU) have significant impact on sleep. Poor sleep is common in this setting, can persist beyond acute critical illness, and is associated with increased morbidity and mortality. In the past 5 years, intensive care clinical practice guidelines have directed more focus on sleep and circadian disruption, spurring new initiatives to study and improve sleep complications in the critically ill. The global SARS-COV-2 (COVID-19) pandemic and dramatic spikes in patients requiring ICU level care also brought augmented levels of sleep disruption, the understanding of which continues to evolve. This review aims to summarize existing literature on sleep and critical illness and briefly discuss future directions in the field.

## Introduction

Adequate sleep is essential for proper immune function, cardiovascular health, optimal cognition and memory, and metabolism, while inadequate sleep has been tied to increased mortality (1–3). Numerous studies over the past 5 years have confirmed this finding, as well as an association between poor sleep quality with incidence of cancer, cardiovascular diseases such as hypertension and diabetes, frailty, and mental health disorders (4–8). The 2018 update to the Society of Critical Care Medicine Clinical Practice Guidelines for the Prevention and Management of Pain, Agitation/Sedation, Delirium, Immobility, and Sleep Disruption in Adult Patients in the ICU (PADIS), added immobility and sleep disruption to their guidelines, as these are considered to be modifiable risk factors for poor ICU outcomes (9). With improved survival of previously highly fatal critical conditions, research has expanded to assess how poor sleep can impact ICU morbidity and mortality metrics. However, existing literature remains limited in delineating how poor sleep in the ICU can be defined, how it may impact patient outcomes, and how sleep quality can be optimized. This review aims to describe the changes seen in the sleep architecture of critically ill patients, discuss the interplay of clinical and physiologic facets of critical illness, including the post-ICU state and sleep, as well as summarize interventions that have been proposed to address sleep disruption in the ICU.

## Sleep architecture and background

In mammals, the inclination to sleep is directed by interaction of homeostatic and circadian mechanisms, otherwise known as the Borbely Model (10). The suprachiasmatic nucleus (SCN) of the hypothalamus controls the circadian clock component by dictating oscillations of gene expression according to an intrinsic time period which is approximately 24.2 h (11). The ability of the circadian rhythm timing to fluctuate allows for zeitgebers (or synchronizers) to adapt the clock to daily behaviors and physiological processes (12). The circadian rhythm not only controls the sleep–wake cycle, but also plays a role stress hormone release, glucose homeostasis, cardiovascular health, body-temperature regulation, and immune response (13). For example, a recent randomized control trial found that early morning vaccination enhanced the immune response to the influenza vaccine compared to afternoon vaccinations in the elderly population (14). The homeostatic component of sleep regulation corresponds to the physical need for sleep. This ‘sleep pressure’ is impacted by the previous night’s sleep quality and quantity, and the cumulative wake time throughout the day (15). In addition to the circadian and homeostatic mechanisms, peripheral clocks have been identified in extra-cerebral tissues such as liver, skin, heart, kidney, and retina, and are capable of acting separately of the circadian rhythm of the SCN (16).

The light–dark cycle is the principal driver, or zeitgeber, in adapting the circadian rhythm. Retinal ganglion cells (RGCs) receive input from environmental light via direct expression of the photopigment melanopsin or secondarily from rods and cones. This information is conveyed to the SCN via the retinohypothalamic tract or directly to other brain regions involved in sleep and mood regulation (17). SCN neural activity is adjusted by the input from light and other zeitgebers, and communicates this information to other areas of the body, including the locus coeruleus-norepinephrine system and the raphe nuclei, which in turn regulate secretion of wakefulness promoting serotonin, norepinephrine, and activity of melatonin (18, 19).

In humans, melatonin is secreted in the dark phase of the circadian rhythm. Sleep proclivity increases about 2 h after this begins. Exposure to light during the day, especially of a certain intensity or in an earlier time period, synchronizes the circadian rhythm and promotes earlier and longer sleep that evening. This is termed a “phase-advance.” Light exposure in the late afternoon or at night effectively prevents the secretion of melatonin from the pineal gland during that time period. This results in a “phase-delay” (16). Blue light is the most efficient wavelength of light at suppressing melatonin release, perhaps due to the high degree of sensitivity that melanopsin containing RGCs have to this facet of light (20, 21).

### Sleep stages in healthy individuals

The normal sleep scoring system for healthy individuals set out by Rechtschaffen and Kales (R and K) in 1968 and subsequently adapted for the American Academy of Sleep Medicine (AASM) guidelines does not apply to critically ill patients (22, 23). The current AASM classification divides sleep into three non-rapid eye movement (NREM) stages and one rapid eye movement (REM) sleep with organized and cyclical transitioning between each stage. Stage 1 (N1) sleep is light sleep characterized by low-voltage, fast electroencephalogram (EEG) activity, typically of short duration. Stage 2 (N2) sleep is distinguished by the presence of K complexes and sleep spindles over a background of typical theta activity. Stage 3 (N3) sleep is also termed deep sleep or slow wave sleep (SWS). This AASM stage includes both stages 3 and 4 of the traditional (R and K) scoring system. N3 sleep is characterized by slow and high amplitude waves with a reduction in muscle tone. This is typically the most restorative sleep and diminishes with age. Finally, stage REM sleep features low-amplitude, mixed-frequency EEG waves, muscle atonia maintained through neuronal circuits in the brainstem, and the hallmark rapid eye movements seen in the electrooculogram (24–26). REM sleep is associated with dreaming, is physiologically distinct from other sleep stages, and is thought to play a crucial role in learning and memory consolidation (27, 28).

### Non-modifiable risk factors for circadian disturbances

Circadian patterns are influenced by nonmodifiable risk factors such as age and gender. For example, healthy women typically have an earlier entrained circadian phase compared to healthy men of the same age, which may contribute to the observed increased prevalence of sleep disorders in women (29–31). Perhaps more pertinent to the scope of this review, due to the prevalence of older adults in our intensive care units, is the circadian misalignment seen with increasing age. Those in later life have lower body temperature amplitudes and decline in melatonin release, possibly contributing to the changes in sleep architecture seen in the aging population (32). However, it is difficult to separate sleep changes that occur with aging from the concomitant effects of certain disease states and medications that are often added in this age group.

Total sleep time (TST) and sleep efficiency steadily decrease with age in healthy adults, accompanied by increased sleep latency and increased nighttime awakenings. The percentage of both stage 1 and stage 2 sleep increase and the percentage of slow wave and REM sleep decrease with age (33, 34). Of note, these are similar to findings seen in critically ill patients. Additionally, in older adults the circadian phase is known to move earlier, or “phase advance,” resulting in peak sleepiness occurring earlier in the evening (35, 36).

Circadian disturbances are associated with increased incidence of cognitive decline and development of neurocognitive disorders. These data are limited to prospective observational studies (37–39). Lower amplitude and phase-delayed circadian body temperature are separately correlated with sundowning syndromes in those with Alzheimer’s disease (40). Increasing amounts of research supports the connection between sleep quality and cognitive dysfunction in older adults. It remains unclear whether these circadian alterations are causal or an effect of disorders of cognitive impairment.

## Critical illness and sleep

**Figure 1:**
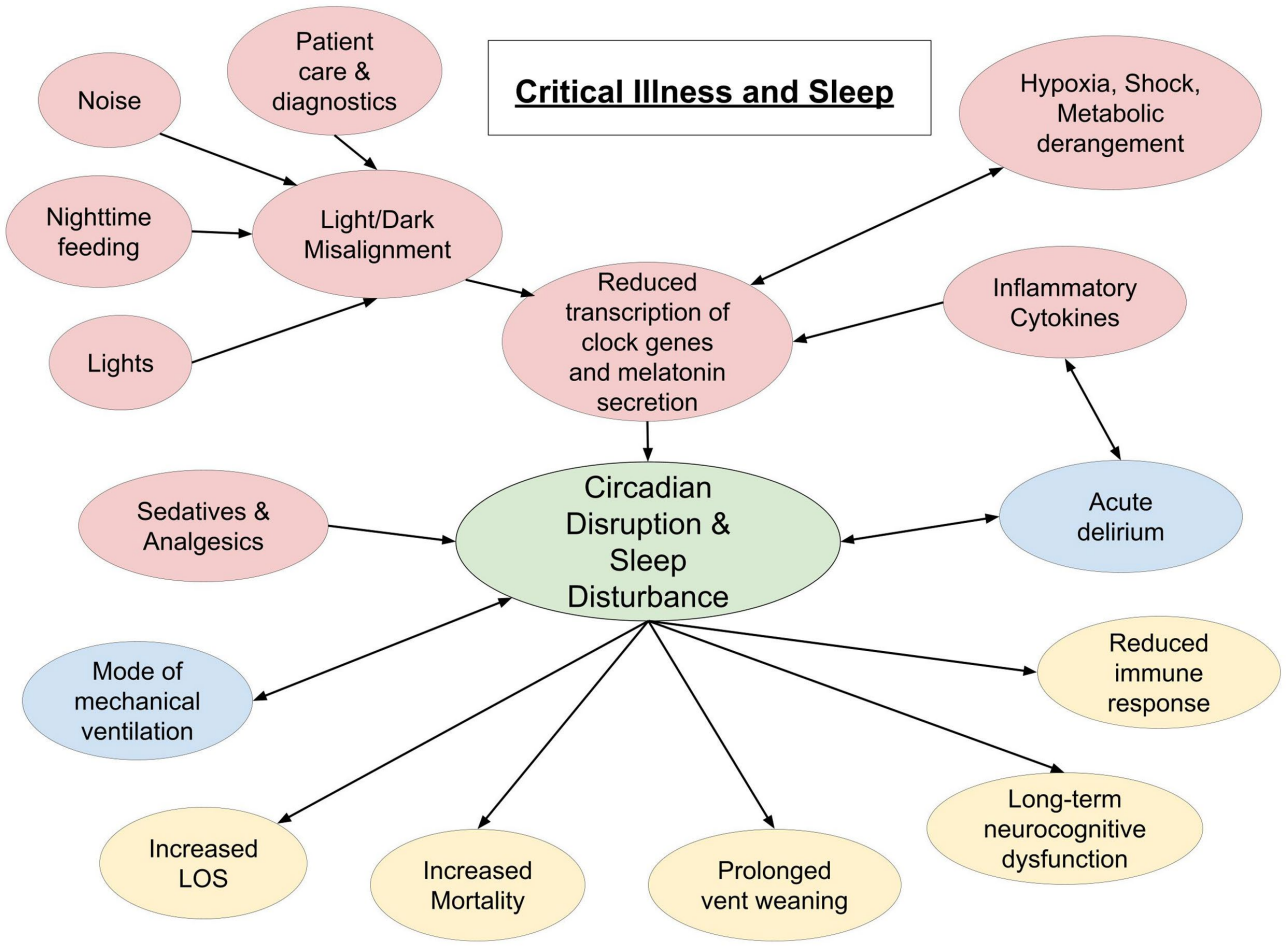
Schematic representation of interplay between critical illness and sleep. Red circles indicate a cause of sleep disruption. Blue circles indicate a bidirectional relationship. Yellow circles represent potential effects of sleep disruption in the critically ill (LOS, length of stay).

There is an involved relationship between critical illness and quality and quantity of sleep. As mentioned above, inadequate sleep is associated with several undesirable health outcomes, including immune dysregulation, that make one susceptible to severe illness. In turn, critical illness, and the medications and interventions that come with it, causes profound disturbances in quality and quantity of restorative sleep [(41); Figure 1]. Given the complex nature of diseases and therapy, there are some limitations to collecting sleep data on critically ill patients.

### Objective sleep evaluation- limitations in the critically ill patient

The gold standard for clinical evaluation of sleep and sleep disordered breathing remains polysomnography (PSG). However, implementing PSG lacks practicality in many situations and has several limitations in the critically ill population, such that the PADIS 2018 guidelines discourage its use in identifying sleep disorders in the ICU (9). Actigraphy, a non-invasive device typically worn on the wrist that measures movement as a surrogate for sleep/wake cycles, is helpful in diagnosing sleep disorders in outpatients, but overestimates sleep time and efficiency in those mechanically ventilated and sedated (42). Single-lead EEG, multi-lead EEG, subjective recall, functional magnetic resonance imaging (MRI), and circadian biomarker measurement have all been studied, but remain impractical or imperfect in the ICU setting (43). Bispectral index analysis (BIS) processes electric activity from the brain and is typically used to monitor depth of anesthesia in the operating rooms and ICU. BIS is effective at distinguishing sleep stages in outpatient healthy volunteers (44). However, this metric is unreliable in identifying sleep stages in ICU patients and thus is not currently recommended (45–47). More recently, an automated scoring algorithm using EEG analysis has shown promise in real-time differentiation of wakefulness from sleep in non-sedated, intubated patients (48).

### Sleep in the critically ill

ICU PSG studies that have been performed show a constant theme of excessive sleep fragmentation, decreased sleep efficiency, increased proportion of stage N1 sleep, and paucity of REM and slow wave sleep (N3). There is heterogeneity of findings regarding whether TST in the critically ill patient is preserved or decreased (49). Possibly, this is due to difficulty distinguishing sleep in the ICU using the standard PSG, or the widely differing indications for and complications of ICU level care (including sepsis, use of mechanical ventilation, and pain) that have their own impact on sleep architecture (50). When TST is preserved, this is due to a large percentage (up to 41%) of sleep occurring in the daytime hours (41, 51). While loss of deep sleep and REM stages is also associated with medications frequently administered in the ICU (such as sedatives, analgesics, and pressors), these findings persist in those who have been off sedation for an average of 3 days (52). Even patients without extreme illness (Acute Physiologic and Chronic Health Evaluation Score II of nine or less), who are sedative naïve (53), and non-ventilated requiring ICU care are noted to have decreased sleep efficiency with frequent cortical arousals, relatively increased stage 1 sleep, decreased N2 and N3, and absence of REM sleep (54).

Light and noise are perhaps the most readily modifiable causes of sleep disruption in the ICU. However, a regression model developed to examine the common drivers of sleep disruption in the critically ill found that above World Health Organization recommended levels of light and sound could only account for about 25% of observed sleep variance (55). Indeed, less easily remedied factors such as pain, attachment to life-sustaining devices, anxiety/stress, and emergent procedures are frequently cited as additional contributors to poor sleep (56, 57).

Critical illness itself is also a major contributor to disrupted sleep. Rhythmic melatonin secretion, a major component of the sleep/wake cycle, is nearly abolished in critically ill patients (58–63). In addition, expression of clock-related genes that maintain circadian patterns are substantially disrupted in patients with septic shock compared to healthy controls (64). There is data to support that inflammatory responses to critical illness, including shock and trauma, can cause circadian remodeling and dyssynchrony secondary to attenuation of clock gene transcriptions when energy is spent on more critical needs (61, 65–67). Vasopressor infusions, commonly required in the ICU, are additionally associated with reductions in REM and SWS (68). This may be secondary to norepinephrine release from the locus coeruleus contributing to increased wakefulness (19).

### Scoring sleep in the critically ill patient

The AASM classification system for sleep is validated for use in healthy patients without neurologic abnormalities, not under the influence of sedatives or analgesics, and less applicable to critically ill patients. Alternate classification strategies have thus been proposed. Drouot, et al., first adapted the staging for sleep in the critically ill in 2012, after previous studies cited difficulty classifying sleep stages by the existing criteria (51, 69). This scoring system is adapted from a cohort of 57 ICU patients admitted with respiratory failure requiring either non-invasive ventilation (NIV) or invasive mechanical ventilation but tolerating pressure support trials, all of whom were off sedation for more than 48 h (70). From this cohort the terms “atypical sleep” and “pathologic wakefulness” were established. These phenomena of abnormal sleep patterns, to be described subsequently, were seen in nearly a third of their cohort.

Watson and colleagues expanded on this pilot scoring system by further defining the phases of “atypical sleep” and applying these criteria to those who were characterized as more critically ill. These patients were mechanically ventilated and, of note, most of the time, sedated (71). In this cohort, 36 of 37 patients were identified as having an atypical sleep pattern, characterized by the lack of sleep spindles and K complexes that traditionally differentiate Stage 2 from other stages of NREM sleep (70).

EEG patterns seen during behaviorally diagnosed wakefulness are also abnormal in critically ill patients. This “pathologic wakefulness” is characterized by a prevalence of slow wave (theta and delta) activity and the absence of normal EEG response to eye-opening (70, 72). The difficulty in differentiating pathologic wakefulness from NREM likely contributes to the overestimation of TST seen in many PSG studies on critically ill patients (49).

These described abnormalities in sleep architecture, when present, are linked to mortality in critically ill patients. One study of 52 ICU patients on mechanical ventilation but not receiving continuous sedation found that PSGs with absent sleep spindles correlated with a significantly increased risk of in-hospital and 90-day mortality. The absence of K-complexes and REM sleep had a trend of increased mortality in this population but did not reach statistical significance (73). Another retrospective study in patients receiving EEG for a diagnosis of delirium showed that those with K-complexes had a reduced odds ratio for death during hospitalization and a significantly reduced mean ICU length of stay. The loss of these N2 features on EEG was associated with a higher degree of encephalopathy (74). A limitation of this study, however, was the use of sedative medications in around two thirds of participants.

The presence of atypical sleep patterns may also predict unfavorable respiratory outcomes. A cohort of patients admitted with hypercapnic respiratory failure requiring NIV were assessed with PSG between nights two and four on NIV. Abnormal EEG patterns, defined in the study as the lack of typical signs of wakefulness and sleep, were predominantly seen in those patients who eventually failed NIV, required endotracheal intubation, or died, while those with normal EEG patterns of sleep and wakefulness were more likely to improve with NIV (69).

### Sedatives and analgesics

In evaluating sleep function in the ICU, one must account for confounders such as intravenous sedatives and analgesics (75). While the effects of these medications appear externally similar to sleep, this induced state in fact differs physiologically from natural sleep. EEG changes from sedative/anesthetic agents are class-specific and dose-specific. The most prominent shared feature between anesthesia and natural sleep is slow-delta wave oscillations (76). As a group, sedating medications are a nidus for sleep disruption in the ICU. The effects of specific classes of medications are discussed below.

Gamma-aminobutyric acid (GABA) stimulating medications such as benzodiazepines lead to a decrease in stage 1, 3, and REM sleep, and thus less restorative sleep for the brain. With the relative increase in stage 2 sleep, however, patients who are dependent on these medications in the outpatient setting may have a perceived improvement in sleep quality due to a decrease in micro-awakenings in this stage from an increased arousal threshold (77). Propofol similarly reduces N3 and REM sleep (78, 79).

Dexmedetomidine is a medication with great promise in the ICU of late, and the highlight of many recent trials (to be discussed further below). Dexmedetomidine is a centrally acting alpha-2 adrenergic agonist that inhibits the release of norepinephrine from the locus coeruleus, which results in the production of a sleep-like state similar to N2 and N3 sleep (80–82).

Data on the effects of opioids on sleep are variable, likely due to dose dependence and the impact of drug dependency versus the drug withdrawal cycle. The consensus is that opioid infusions reduce N3 and REM sleep and decrease TST in the ICU (83).

Another well-described effect of opioid treatment is the development of sleep apnea, predominately due to effects on the central nervous system, leading to central sleep apnea (CSA) (84). More recent trials suggest that administration of opioids during mechanical ventilation is also linked to increased upper airway (UA) collapsibility both during wakefulness and during sleep (85). In a group of patients with low pre-ICU risk of obstructive sleep apnea (OSA), 71% of patients developed OSA the night after extubation. There is a dose dependent relationship between the severity of OSA by Apnea-Hypopnea-Index (AHI) and the dose of opioid the ICU patients received in the 24 h prior to extubation (83). UA collapsibility is the most likely mechanism of this finding and is thought to be due to opioid-induced suppression at the hypoglossal motor nucleus, activity of which is necessary for maintaining airway patency (86). In addition, opioids increase the arousal threshold, blunting the response to obstructive events (87, 88). Interestingly, optimizing volume status may be a way to mitigate the risks of OSA in critically ill patients, especially those receiving opiates/sedatives, as jugular vein volume and UA mucosal water content (as measured by MRI) are associated with increase in OSA severity (89). However, there is no data yet evaluating increased need for re-intubation in those who develop UA collapsibility and OSA. This would be of particular interest when considering the associated effects of respiratory failure on sleep, as reviewed in the following section.

### Sleep and respiratory failure

Mechanical ventilation and the concomitant use of sedatives/analgesics impacts sleep quality in the ICU. In a study of recently extubated patients, it was determined that total sleep time and sleep efficiency were significantly decreased when compared to that in the intubated state (90). In this study, though intubated patients had increased TST, the sleep was more likely to be atypical, which may be considered a marker of critical illness severity. The improved sleep metrics in the intubated population could be due to optimization of gas exchange and work of breathing (91), the effect of sedation, or by reduced effects of concomitant sleep disordered breathing.

It is likely that the mode of ventilation chosen for continuous mechanical ventilation impacts sleep quality as well. An early study by Parthasarathy and Tobin concluded that patients receiving high-level pressure support ventilation (PSV) without a backup rate (average pressure support of 16.8 cmH20) compared to assist control ventilation (ACV) settings had significant central sleep apneas resulting in sleep fragmentation (92). They found a 23 and 30% increase in inspiratory and expiratory time, respectively, along with a 33% decrease in respiratory rate on PSV. The increased inspiratory time led to an increase in tidal volume during sleep and resultant hypocapnia below the apnea threshold (93). It is likely that the set pressure support was greater than necessary given the decrease in oxygen consumption and carbon dioxide (CO_2_) production during sleep, which results in decreased minute ventilation (9). Indeed, the addition of dead space to the ventilator circuit reduced the frequency of apneas and sleep fragmentation observed in these patients. Limitations of this study, however, were the high percentage of patients with congestive heart failure and the lack of exclusion of sedative agents.

One other study conducted by Cabello and colleagues documented central sleep apneas in PSV compared to ACV. They noted central apneas in 9 of 15 patients receiving either physician adjusted PSV or automatically adjusted PSV, but observed a similar level of sleep fragmentation and arousals compared to ACV. Less than 10% of sleep fragmentation was therefore attributed to the CSA events. The patients in all groups had similar tidal volume and minute ventilation but the PSV groups were adjusted to an average inspiratory pressure of 14–16 cmH20 (94). This again raises the question whether higher inspiratory pressures may have been a cause of central events.

In another study of patients intubated for primary pulmonary issues, a lower-level pressure support (6 cmH20 inspiratory pressure) was compared to ACV in the weaning period. Those receiving ACV had improved sleep quality and sleep duration in all stages, particularly the restorative sleep stages. Sleep disruption seen with PSV could not be attributed to central sleep apneas as there were no recorded apneas in any group (95). A parallel study from the same group comparing low level PSV to pressure control ventilation (PCV) similarly saw increased sleep efficiency and improved percentages of sleep stages, including REM, in the PCV group compared to the PSV group. Again, no apneas were seen, likely due to the lower tidal volumes achieved on low-level PSV (96). Sustained respiratory muscle efforts required during pressure support ventilation could contribute to the reduced sleep parameters observed on this mode.

Roche-Campo et al. examined patients who required a tracheostomy for prolonged weaning from mechanical ventilation (2–3 weeks). These sedation-free patients were randomized to spontaneous ventilation (trach collar) or mechanical ventilation with pressure support. Of note, this cohort of patients had variable reasons for admission, the most common ones being cardiac surgery, cardiac failure, pneumonia, and pancreatitis. In accordance with existing data on sleep in the ICU, there was a high amount of sleep fragmentation, low percentage of REM, and reduced sleep efficiency in both groups. Use of mechanical ventilation had no impact on sleep quality in these weaning patients (97).

Importantly, in patients deemed ready to be weaned from mechanical ventilation, those with atypical sleep or lack of slow wave sleep or REM sleep are more likely to require a prolonged wean compared to those with normal sleep patterns (5 days vs. 2 days). Furthermore, patients with more atypical sleep patterns had a longer duration of mechanical ventilation and required more sedation prior to their PSGs (98). A similar study showed that the amount of time spent in full (non-pathologic) wakefulness is associated with a successful Spontaneous Breathing Trial (SBT) and extubation (72).

To better understand the association between altered sleep and liberation from the mechanical ventilator, Rault and colleagues investigated the effects of one night of sleep deprivation on healthy subjects’ respiratory endurance. They found that one night of sleep deprivation resulted in a 50% reduction in inspiratory muscle endurance, an 11.1% decrease in maximum tidal volume, and a 25.9% decrease in diaphragmatic electromyography (EMG) activity (99, 100). This study supports the idea that sleep deprivation itself contributes to clinically significant neuromuscular weakness that may manifest as difficulty weaning from mechanical ventilation.

Focusing more efforts on optimizing sleep efficiency in the critically ill population may therefore reduce time needed to wean from mechanical ventilation, reduce total ventilator days, ICU length of stay, and hospitalization costs.

## Sleep and its effects on cognitive function and delirium

Delirium, a disturbed level of consciousness characterized by fluctuating inattention and agitation, is associated with numerous poor outcomes, including mortality, morbidity, length of stay, and health care costs (101). Thus, many efforts have gone into trying to reduce the burden of delirium throughout hospital stays, with promotion of adequate sleep at the forefront.

The relationship between sleep and altered cognition is complex and likely bidirectional. Reduced REM and slow wave sleep are considered either a contributor to delirium or a manifestation of delirium (102, 103). There is a growing body of evidence that poor sleep precedes the development of cognitive impairment (104, 105). With several prospective trials over the past several years, we have gained much knowledge on acute delirium in the critically ill, but still cannot say for certain if poor sleep is the causative factor.

As described by a recent prospective cohort study, ICU patients with delirium, in comparison to those without delirium, have even more profound reductions in REM sleep. This data comes from PSG recordings over one 24-h period of ICU patients. Delirium was diagnosed using the Confusion Assessment Method (CAM-ICU) scale and those with delirium were matched with patients of similar ages and admission diagnoses without delirium. In addition to reduced REM sleep and slow wave sleep in patients with delirium, melatonin levels were significantly lower and cortisol levels significantly higher in this population. A limitation to this study was that the patients with delirium were found to have higher APACHE II scores, possibly confounding results (106).

While these data are promising in defining the relationship between sleep and delirium in the ICU, robust studies showing direct causal relationship between poor sleep and cognitive dysfunction remain lacking (107). One prospective study in post-operative patients that analyzed EEGs in all post-surgical patients and later screened for delirium found that those diagnosed with delirium later in the hospital stay had a significant reduction in TST and 0% REM sleep both night-one and night-two post-operatively. While this may be a sign that sleep deficiency leads to delirium, there is no way to prove that this absence of adequate sleep is not merely a manifestation of developing delirium (108).

As mentioned in the previous section, sedation is linked to development of OSA in extubated critically ill patients. OSA is a risk factor for delirium, though the exact mechanisms behind this are not yet clear. One possibility is the augmentation of inflammatory cytokines as a result of cyclical desaturations from obstructive respiratory events. These hypoxemic events decrease cerebral oxygen delivery and alter brain energy metabolism, contributing to cognitive dysfunction and EEG slowing (109, 110). Increased inflammatory cytokines from critical illness or sepsis are similarly correlated with an increased risk of delirium (111, 112).

ICU delirium is not just associated with poor hospital outcomes, but also has long term health consequences. Longer duration of hypoxia induced delirium, in addition to delirium attributed to severe sepsis or sedative administration, has been associated with more severe cognitive impairment at 1 year follow up (113, 114). These prospective studies utilized the Repeatable Battery for the Assessment of Neuropsychological Status (RBANS) test, a self-administered test measuring attention, memory, and visuospatial abilities typically employed in the diagnosis of dementia (115).

To further elucidate the connection between sleep fragmentation and cognitive dysfunction, Wilcox and colleagues performed actigraphy and RBANS testing on a cohort of critically ill patients who required at least 3 days of mechanical ventilation at 7 days, 6 months, and 12 months post discharge. They found a statistically significant correlation between sleep fragmentation seen at 7 days and cognitive dysfunction. No other actigraphy-estimated sleep disturbance was associated with cognitive impairment at the 6- or 12-month mark (116). These findings are limited by the small sample size at later follow ups (due to deaths and withdrawals from the study) and use of actigraphy rather than polysomnography.

## Post-intensive care syndrome and sleep

Improved survival over the past several decades of many critical illnesses has led to more focus on the long-term impact that ICU stays have on patients and families. The high prevalence of numerous morbidities after critical illness has led to the term “post-intensive care syndrome” or PICS. PICS is defined as any new or worsening impairments in physical, mental, or cognitive health that persist after critical illness (117).

PICS is extremely common. A recent multicenter cohort reports the prevalence of PICS in several different cohorts. In a group of critically ill patients admitted to the ICU prior to the SARS-Cov-2 pandemic, the prevalence of PICS at 12 months was 39%. In non-COVID infected patients admitted to the ICU during the COVID pandemic, the prevalence was 53% at 12 months follow up. The increase in PICS in this population was attributed to worsening symptoms in the cognitive and mental health domains (118). This suggests that stressors of the pandemic may have adversely influenced patients’ recovery from their critical illness. The most common symptoms of PICS include depressed mood, memory disturbances, weakness or decreased mobility, and sleep disturbances (119).

Sleep disturbances are present in over 50% of polled ICU patients 1 month after discharge (120), and this prevalence remains elevated at least until 12 months after an ICU stay, which is the longest follow up described to date (121). The degree of delirium experienced during the hospitalization may play a role in these later symptoms. In a cohort of ICU patients 1 year after ICU discharge, total days of ICU delirium was significantly associated with increased sleep disturbances and a trend toward increased disability (122).

In polysomnography performed after critical illness, the abnormalities seen during the critically-ill period often persist: high percentage of N1 sleep, lack of slow wave and REM sleep. At six-month follow up, there is some increase in slow wave sleep, but REM sleep is continually lacking, at only 9% of TST. An additional finding on PSG post-ICU is an increased prevalence of sleep apnea compared to the general population (123, 124). This complex relationship is to be discussed in a subsequent section.

## SARS-Cov-2 acute illness and sleep

Similar to all ICU patients, self-reported assessments indicate that patients admitted with mild-to-moderate, or even severe COVID-19 requiring prolonged mechanical ventilation continue to have poor sleep after hospitalization, with the highest rates cited at 75% at 3 month follow up (125–127). In fact, sleep difficulty is among the most common complaints (after fatigue) at 6-month follow up after COVID (128).

A cohort of 172 COVID-19 ARDS survivors underwent actigraphy 3 months after their ICU stay for objective analysis of sleep quality. Patient subjective reports correlated closely with actigraphy findings. Actigraphy confirmed that a majority of patients had reduced TST and sleep efficiency, high amounts of wakefulness after sleep onset (WASO), and evidence of continued circadian misalignment at the 3-month follow up (129). Subsequent evaluation at 6 months showed an association between length of stay in the hospital, ICU length of stay, use of mechanical ventilation, and the degree of circadian fragmentation. Body Mass Index (BMI) at baseline (prior to infection) also correlated with significantly worsened sleep quality and circadian misalignment (130). These results are similar to studies performed on ARDS survivors pre-pandemic, where actigraphy showed altered circadian alignment and reduced sleep efficiency (131).

One of the few groups that have thus far performed polysomnography on COVID-19 survivors studied patients with COVID induced ARDS 1 month after discharge. There was reduced total sleep time and sleep efficiency, as well as reduced deep sleep at only 8% of TST. REM sleep was only slightly depressed in this population at 19% of TST, which is notably a markedly increased percentage than seen in other, non-COVID, studies. While the APACHE II scores were not indicated in this study, it is possible that the subjects were less critically ill given the low requirement for invasive mechanical ventilation in the cohort. Most remarkably, perhaps, this study identified obstructive sleep apnea in a majority of the post-ICU population (132), similar to the data reported in non-COVID PICS (124).

## Sleep apnea syndromes and critical illness

Obstructive sleep apnea is a common medical comorbidity and is associated with medical conditions that frequently contribute to ICU admission, such as obesity, heart disease, diabetes, and older age (133). While sleep apnea syndromes may be a risk factor for critical illness, a causal relationship has yet to be defined. So far, literature reports a preponderance of sleep apnea in the post-ICU period. However, whether this process was present before or developed after ICU admission is yet to be elucidated.

One study by Alexopoulou et al. showed that 29 out of 36 subjects (80.6%) who were intubated and mechanically ventilated for Acute Respiratory Distress Syndrome (ARDS) met a diagnosis of OSA (based on AHI ≥ 5 events per hour) 10 days after discharge from the ICU. Sixty one percent of these subjects had moderate OSA with an AHI ≥ 15 events per hour. While the majority had a predominance of obstructive apneas, several patients had mixed central and obstructive apneas, with one patient having strictly central apneas. Seventy-two percent of subjects continued to have at least mild-OSA at 6 months post-discharge (124). These findings contrast with a study by Dhooria and colleagues, which showed only a 15% prevalence of OSA at 1 month after discharge from the ICU (134). This cohort, while still predominantly with a ICU diagnosis of ARDS, were significantly younger than the previous study, with an average age of only 24 compared to 54 years in the Alexopoulou cohort.

Similar to Alexopoulou’s group, Goyal et al. reported high rates (73%) of moderate-to-severe OSA in a group of COVID-19 ARDS survivors (132). OSA is frequently cited as a risk factor for severe COVID disease (135–139), though much of this data comes from retrospective, patient reported metrics (140). This association may be confounded by other independent risk factors such as BMI (141, 142). Larger prospective trials and mechanistic studies are necessary to understand whether OSA has an independent, direct relation to COVID severity or if incidence of sleep apnea is intensified by COVID-19 infection (143, 144).

OSA may separately predispose patients to symptoms of Persistent Acute Sequelae of SARS-Cov-2, or PASC. A cohort of 60 critically ill patients with prior COVID were analyzed with home sleep apnea testing and actigraphy. Over 60% of the cohort were identified as having at least mild OSA as measured by Respiratory Disturbance Index (RDI). The authors of this study consider these patients to have had undiagnosed OSA prior to their admission for COVID. They found that OSA patients were more likely to have cognitive impairment at 1 year follow up (145). PASC and OSA have similar presentations with fatigue, excessive daytime sleepiness, and depression. OSA should perhaps then be diagnosed and treated with Continuous Positive Airway Pressure (CPAP) prior to making a formal diagnosis of PASC.

**Figure 2:**
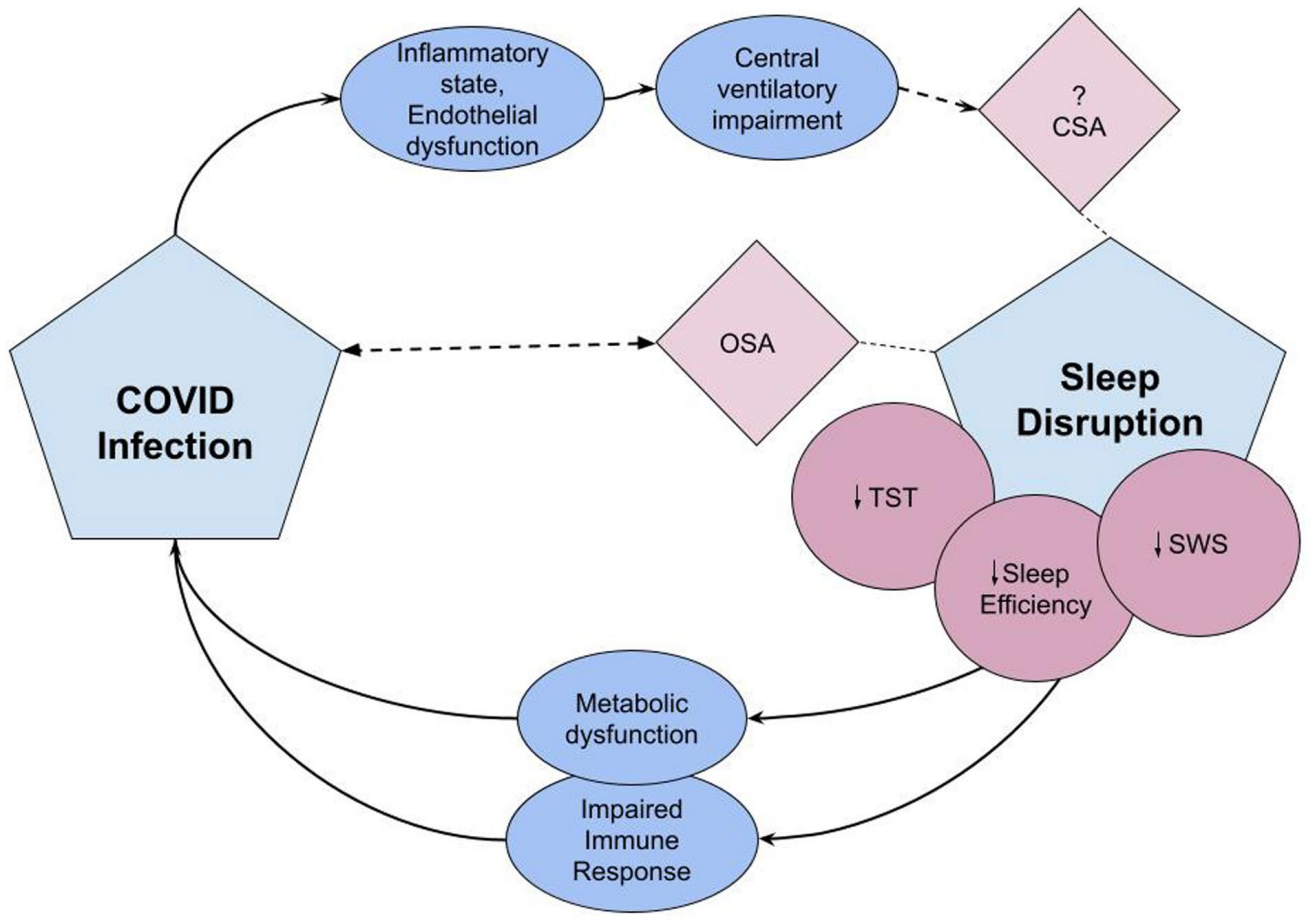
The pathophysiologic effects of sleep disruption on risk for COVID infection and COVID’s effects on sleep disruption. Covid infection induces an inflammatory state and endothelial dysfunction leading to central ventilatory impairment, potentially triggering central sleep apnea syndrome (152). Sleep disruption associated with critical COVID infection includes reduced TST, sleep efficiency, and reduction in SWS (132). Lack of adequate sleep causes immune and metabolic dysfunction which may predispose to viral infections and impact response to immunizations (14). OSA is associated with COVID-19 infection. Either OSA is a risk factor or undiagnosed and unmasked by COVID infection (143). CSA, central sleep apnea; OSA, obstructive sleep apnea; TST, total sleep time; SWS, slow wave sleep.

COVID infection may additionally contribute to sleep disorders through involvement of the central nervous system. In 11 patients with sleep-related complaints after COVID infection, PSGs showed REM without atonia in over one third of subjects (146). Literature also alludes to a prevalence of CSA in the setting of COVID infection. While in the prospective study noting this, the patients all had cardiac risk factors that may have predisposed to CSA (147), there are case reports of CSA with periodic breathing after COVID-infection seen in the absence of typical risk factors or medications (148–150). The SARS Cov-2 virus is theorized to cause central nervous system (CNS) dysfunction through direct infection of the nervous system, induction of a hypercoagulable state, or immune-mediated inflammatory processes [(151, 152); Figure 2].

Research on sleep disorders and their complex relationship to COVID-19 is still in the beginning stages. Larger, prospective trials are needed.

## Improving sleep in the ICU

Restorative sleep is essential to recover from acute illness. Numerous pharmacologic and non-pharmacologic interventions have been tested to improve sleep quality in intensive care units with varying levels of success (Table 1).

**Table 1:** Non-pharmacologic and pharmacologic ways to improve sleep in the ICU.


*Nonpharmacologic*	
Light–dark alignment	Minimize overnight light exposure Eye Masks
Noise disruptions	Ear plugs Minimize non-essential care tasks at nighttime hours, machine alarms
Reduce psychological distress	Control pain and anxiety Avoid deliriogenic medications (GABA agonists, antihistamines, opioids, corticosteroids) Monitoring and avoiding drug withdrawal
*Pharmacologic*	
Melatonin	May improve sleep duration and efficiency [10 mg dosing administered at 9 PM (153)] No effect on delirium
Melatonin receptor agonists	No proven effect on sleep May reduce delirium [8 mg dosing (154)]
Dexmedetomidine	Titrated to a RASS of −1 to −2 increases TST and sleep efficiency [Dosing 0.1–1.4 μg/kg/h (155)] Decrease in delirium Most supported in literature but not yet recommended by SCCM

GABA, γ-Aminobutyric acid; RASS, Richmond Agitation Sedation Scale; TST, total sleep time; SCCM, Society of Critical Care Medicine.

### Non-pharmacologic interventions

Non-pharmacologic interventions with documented success include eye masks and ear plugs (156), interactive music therapy (157), aromatherapy (158), and multi-disciplinary bundles (9). Multidisciplinary bundles have demonstrated significant and clinically meaningful improvements in several core ICU outcomes, such as survival, length of stay, readmission, mechanical ventilation use, and delirium (159, 160). When light and noise optimization were incorporated into a pre-existing ICU bundle, sleep quality was improved in a cohort that excluded patients with cognitive dysfunction at baseline or delirium (161). After the update of the 2018 PADIS guidelines, quality improvement initiatives have more thoroughly incorporated Immobility and Sleep into multi-modal ICU bundles. Interventions predominately focus on minimizing noise, light, and non-essential care activities during the nighttime hours (57, 162).

ICU settings have considerable light–dark misalignment, with high levels of light at night and reduced levels of natural lighting in daytime hours, which can counteract circadian rhythmicity (163, 164). Through standardized strategies to reduce sound and adjust light to optimize circadian rhythm, new ICU protocols have successfully improved sleep quality and shown a trend toward decreasing delirium compared to standard ICU care (165).

### Pharmacologic interventions

Perhaps the most heavily studied pharmacologic therapies for sleep promotion over the past several years are melatonin and dexmedetomidine. As mentioned earlier, dexmedetomidine shows great promise in optimizing sleep in critically ill patients (80–82). PADIS 2018 guidelines currently do not recommend use of dexmedetomidine for sleep promotion in the ICU, citing that no trials at that time had identified an increase in the restorative sleep stages or decrease in sleep fragmentation (9). In a study recording 57 h of continuous polysomnography, dexmedetomidine infusion titrated to a Richmond Agitation Assessment Scale (RASS) of −1 to −2 was associated with increased sleep efficiency and TST and reduced sleep fragmentation when compared to sleep in the same patient prior to its use. There was no effect on SWS or REM, but use of dexmedetomidine helped to shift daytime predominant sleep to nighttime hours (166). In two recently published randomized controlled trials, use of dexmedetomidine has been proven to increase TST, improve sleep efficiency, and in the more recent 2023 study, to significantly reduce the sleep fragmentation index (155, 167). Dexmedetomidine, either independently or via its positive effects on sleep, is similarly associated with decreased prevalence of post-operative delirium (168, 169). Trials to determine the effects of the drug on long-term delirium and cognition are underway (170).

Melatonin’s therapeutic role in sleep disorders remains controversial; it is not recommended by the PADIS or AASM Clinical Practice Guidelines for general use in adults due to weak evidence (9, 171). In the critically ill, however, some trials support the use of melatonin to improve subjective sleep quality (172, 173). Only two small randomized control trials (RCTs) have evaluated the effects of melatonin or melatonin agonists using objective sleep assessments (153, 174). In Shilo’s study, small doses of melatonin increased sleep duration and quality in a cohort of eight ICU patients, but total sleep time achieved, as assessed by actigraphy, remained less than that observed in hospital ward controls. The small sample size, presence of mechanical ventilation in some ICU patients, and short duration of the intervention (3 days) are limiting factors to wider application of these findings. Bournes’ study utilized Bispectral Index to analyze sleep efficiency index and TST with melatonin treatment. Patients randomized to receive 10 mg of melatonin slept 3.5 h nightly compared to 2.5 h in the placebo group and had improved sleep efficiency. Given the small sample size and several missing data points, these findings also have unclear significance.

While melatonin use may have some positive prognostic impact, such as reduction in number of days on mechanical ventilation (175), data on its role in prevention of ICU delirium are heterogeneous. Despite prior positive trials (154, 176), a recently published multi-center randomized trial involving 847 critically ill patients failed to detect a statistically significant effect between melatonin use and reduction of delirium (177).

The melatonin agonist ramelteon, however, has been shown to decrease the occurrence rate and duration of ICU delirium in a small, single center randomized control trial. This trial also noted a trend toward reduction in ICU length of stay and decreased frequency in nighttime awakenings with ramelteon use (154). A previously published multicenter, randomized control trial similarly found a reduction in delirium in elderly admitted patients given ramelteon. Only 24 of the enrolled patients were admitted to the intensive care setting. This trial did not show any differences in sleep metrics (178). Further investigation is needed to more formally evaluate melatonin and melatonin agonists’ impact on sleep quantity and quality in critically ill patients.

Dual orexin receptor antagonists are a relatively new group of medications that promote sleep by blocking the orexin receptor, which normally signals to maintain wakefulness (179). While there is a lack of evidence to support an improvement in sleep quality with orexin receptor inhibitors, several retrospective trials have shown a reduction in rates of delirium in the acute care setting (180, 181). Larger, prospective, randomized, placebo-controlled trials are warranted.

A practical limitation to the use of melatonin, ramelteon, and the orexin receptor antagonists is their strictly oral formulations. These agents would not be applicable to patients with contradictions to oral feeding or enteral access, such as those with bowel obstruction or gastrointestinal bleeding, or patients with poor gut absorption. This may be another area for potential future research and development.

## Discussion

Sleep architecture, duration, and quality are greatly impacted by critical illness and associated complications, including respiratory failure, sepsis, and delirium. Sleep disturbance in the ICU population contributes to mortality and is linked to increased morbidity such as prolonged mechanical ventilation, increased length of stay, and cognitive disturbances that may persist months after ICU discharge. Despite several encouraging trials published recently on the optimization of critical illness-related sleep dysfunction, there remains significant room for continued development. A multidisciplinary group from the American Thoracic Society (ATS) recently convened to discuss future steps in advancing knowledge and outcomes pertaining to sleep and circadian disruption in critical illness. Six key research priorities were declared, focusing on improving measurement of sleep disturbance in the ICU, better defining the relationship between sleep disturbance and patient centered outcomes, achieving sustainable multicomponent interventions for sleep, and maximizing strategies for multi-site, multidisciplinary treatment investigations (182). In addition to the areas identified in the research statement, remaining gaps also include further defining the effect of COVID on sleep and circadian disturbance and building upon our understanding of the complex relationship between sleep disruption and delirium, both of which are linked to poor ICU outcomes and remain challenging areas to navigate in the care of the critically ill.

## Author contributions

EE researched the literature and wrote the review. JW reviewed and edited the work. All authors contributed to the article and approved the submitted version.

## Conflict of interest

The authors declare that the research was conducted in the absence of any commercial or financial relationships that could be construed as a potential conflict of interest.

## Publisher’s note

All claims expressed in this article are solely those of the authors and do not necessarily represent those of their affiliated organizations, or those of the publisher, the editors and the reviewers. Any product that may be evaluated in this article, or claim that may be made by its manufacturer, is not guaranteed or endorsed by the publisher.
